# The cognitive connectome of men and women: a study on sex differences across three cohorts

**DOI:** 10.1186/s13293-026-00866-0

**Published:** 2026-03-14

**Authors:** Daphne Gasparre, Annegret Habich, Lídia Mulet-Pons, Roraima Yanez-Perez, Eric Westman, José Barroso, Lídia Vaque-Alcázar, David Bartres-Faz, Paolo Taurisano, Daniel Ferreira, the Alzheimer’s Disease Neuroimaging Initiative

**Affiliations:** 1 Division of Clinical Geriatrics, Centre for Alzheimer Research, Department of Neurobiology, Care Sciences, and Society, Karolinska Institutet, Stockholm, Sweden; 2 Department of Translational Biomedicine and Neuroscience (DiBraiN), University of Bari Aldo Moro, Bari, Italy; 3 Department of Medicine, Faculty of Medicine and Health Sciences, Institute of Neurosciences, University of Barcelona, Institut d’Investigacions Biomèdiques August Pi i Sunyer (IDIBAPS), Barcelona, Spain; 4 Department of Clinical Psychology, Psychobiology and Methodology, Faculty of Psychology, University of La Laguna, Canary Islands, Spain; 5 Facultad de Ciencias de la Salud, Universidad Fernando Pessoa Canarias, Las Palmas, Spain; 6 Sant Pau Memory Unit, Department of Neurology, Hospital de la Santa Creu i Sant Pau, Biomedical Research Institute Sant Pau, Universitat Autònoma de Barcelona, Barcelona, Spain

**Keywords:** Gender, Healthy aging, Cognitive connectome, Graph theory

## Abstract

**Background:**

Cognitive processes are essential for efficient daily functioning. Demographic factors such as age and education influence cognitive performance. However, the impact of sex on cognition is less understood and previous research has reported inconsistent findings. We investigated sex differences in cognitively unimpaired adults in three cohorts, using two complimentary approaches: a univariate approach to compare direct performance across cognitive domains and the multivariate approach of graph theory to compare global and nodal features as well as the modular organization of cognitive connectomes.

**Methods:**

We included 4,259 cognitively unimpaired participants (334 from the GENIC cohort, 3,703 from the National Alzheimer’s Coordinating Center [NACC], and 222 from the Alzheimer’s Disease Neuroimaging Initiative [ADNI]). Cognitive variables were corrected for age and education, and cognitive connectomes were constructed using Spearman correlation coefficients. Sex differences in cognitive performance were examined through ANCOVAs as well as global and nodal network measures.

**Results:**

Univariate analyses showed significant sex differences in three out of five cognitive domains across cohorts, mainly of small effect sizes. Graph theory analyses revealed minimal sex differences in cognitive module organization and no significant differences on global network measures, except for a higher modularity observed in women compared to men in the NACC. In contrast, nodal analyses revealed sex differences in several network measures.

**Conclusions:**

Sex differences in cognition seem to be of small effect size and limited to specific cognitive domains or cognitive variables, while the overall organization and global features of cognitive connectomes were largely comparable between men and women. Future studies should clarify whether men and women may rely on slightly different cognitive strategies to approach cognitive tasks without overt differences in cognitive ability.

**Supplementary Information:**

The online version contains supplementary material available at 10.1186/s13293-026-00866-0.

## Abstract (plain language summary)

This study explored whether men and women differ in their cognitive abilities. Previous research has suggested that women may perform slightly better on verbal tasks, while men may do better on some attention or visuospatial tasks. However, several studies have only reported small or no differences, and it remains unclear whether cognitive performance does differ between men and women or they basically use different strategies to perform cognitive tasks. To investigate this question, the researchers analyzed data from three international populations of cognitively healthy adults. Participants completed a range of cognitive tests. The research team used two types of analysis: traditional analyses that compare performances on each individual cognitive task, and a newer method called graph theory, which looks at how different cognitive skills are connected and work together as a network, known as the “cognitive connectome.” The results confirmed that, although men and women differed slightly on some specific cognitive tasks, these differences were generally small. Most importantly, the overall organization of the cognitive connectome was highly comparable in men and women. However, when the researchers looked more closely at individual parts of the connectome, they found some subtle differences. These differences suggest that men and women may rely on slightly different cognitive strategies when performing certain tasks, even though they reach a similar overall level of performance. These findings support the idea that men and women are more similar than different in their cognitive abilities.

**Supplementary Information:**

The online version contains supplementary material available at 10.1186/s13293-026-00866-0.

## Highlights

- This study investigated sex differences in cognition using both univariate tests and graph-theory analysis of the “cognitive connectome.”
- Univariate analyses showed that women had a slightly better performance in memory and men a slightly better performance in language and attention tasks, consistently across three cohorts of cognitively healthy adults.
- Graph analyses showed that correlation matrices, modular structure, and global network features of the cognitive connectome were highly similar between men and women.
- Subtle sex differences emerged only at the nodal level of the connectome.
- Results indicate that men and women achieve overall comparable cognitive performance but may use slightly different cognitive strategies.

**Supplementary Information:**

The online version contains supplementary material available at 10.1186/s13293-026-00866-0.

## Background

Cognitive processes are central to human functioning and behavior. These processes encompass a range of functions such as memory, attention, language, problem-solving and decision-making, among others. Several demographic characteristics such as age, sex, and education have an impact on human cognitive processes. For example, as the age increases, cognitive performance declines. The impact of education is more complex. While literacy level does not seem to have a strong effect on rate of cognitive change, a higher educational attainment has been associated with a higher cognitive performance at a given time point ([1–4]).

The impact of sex on cognition remains less clear. Studies using univariate approaches for data analysis have generally reported that women achieve a worse performance than men in visual-spatial working memory tasks, mental rotation and sustained attention tasks ([5–7]). In contrast, women usually achieve higher scores than men in verbal memory tasks, phonemic and semantic fluency, and selective attention ([8–12]). These findings have also received additional support from neuroimaging studies that revealed sex differences in brain activation during the performance of cognitive tasks. For example, in some functional Magnetic Resonance Imaging (fMRI) studies, women exhibited lower activations than men in parietal regions and higher activations in frontal and inferior temporal regions when performing a task of mental rotation and in visuospatial processing [13, 14]. Another study using diffusion tensor imaging reported different brain connectivity patterns in men and women [15]. In particular, women showed lower structural within-hemispherical connectivity, but more interhemispheric connections than men [15]. These differences in functional and structural connectivity could partly underlie the reported sex differences in cognitive performance.

However, several univariate studies have reported that sex differences in cognitive performance often have small effect sizes or are no longer significant after controlling for cofounders such as education ([10, 16, 17]). Furthermore, several univariate studies reported no sex differences in cognition [18–20]. Indeed, Hyde introduced the Gender Similarities Hypothesis, which proposes that men and women have a similar performance on most, though not all, psychological and cognitive variables. That hypothesis is supported by some meta-analyses, although there is recognition that sex differences may exist in a few cognitive processes [21–23].

One potential reason for these inconclusive results on sex differences on cognitive performance is the limited capacity of univariate analysis to capture complex associations among multiple cognitive variables. In this context, graph theory offers the opportunity to comprehensively investigate intricate associations among cognitive variables [24]. The “cognitive connectome” is a novel concept that reflects the structure, organization, and complex interconnections among multiple cognitive variables (Garcia-Cabello et al., [25]). To the best of our knowledge, only four studies have applied graph theory to cognitive data in cognitively unimpaired adults ([24–27]), and none has investigated sex differences on the cognitive connectome yet.

The overall goal of the current study was to investigate sex differences in the cognitive connectome of men and women using graph theory on multiple cognitive variables and compare the findings with those obtained from traditional univariate analyses. We hypothesized that the multivariate approach of graph theory would capture sex differences in cognitive performance beyond those captured by univariate analysis [26]. Specifically, we anticipated no differences between men and women in global features of their cognitive connectomes, as cognitively unimpaired individuals are generally expected to exhibit similar overall cognitive functioning. However, based on previous univariate studies, we expected differences in nodal analysis for specific cognitive tests and associations between variables, reflecting sex differences in cognitive processes [16, 19, 28].

## Methods

### Participants

We used data of cognitively unimpaired individuals from three international cohorts:

GENIC (Group of Neuropsychological Studies of the Canary Islands) [29]: this dataset included 334 individuals aged 37–78 years, 188 women and 146 men.

NACC (National Alzheimer’s Coordinating Center) [30]: this dataset included 3703 participants aged 45–101 years, 2127 women and 1576 men.

ADNI (Alzheimer’s Disease Neuroimaging Initiative, adni.loni.usc.edu): this dataset included 222 individuals aged 60–90 years, 108 women and 114 men. The ADNI was launched in 2003 as a public-private partnership, led by Principal Investigator Michael W. Weiner, MD. The primary goal of ADNI has been to test whether serial MRI, positron emission tomography, other biological markers, and clinical and neuropsychological assessment can be combined to measure the progression of MCI and early Alzheimer’s disease.

Selection criteria for cognitively unimpaired individuals were highly comparable across the three cohorts. Specifically, the three cohorts used extensive clinical evaluation with age-, sex-, and education-adjusted norms for interpretation of cognitive and clinical data, as fully described in ([29, 31]; www.adni-info.org). Despite similar procedures, we clarify some minor differences. All participants from GENIC were right handed while this was not a selection criteria in NACC or ADNI (however, the frequency of left handed participants in the source population is low). In all three cohorts, participants with a clinical history of neurological or psychiatric disorders, history of substance abuse, uncorrected vision or hearing issues, and/or systemic health conditions with a potential impact on cognitive performance, were excluded. Additionally, the GENIC cohort specifically excluded individuals with brain tumors and/or hippocampal sclerosis in MRI [25, 27]. Therefore, because of the overall comparable criteria for selection of cognitively unimpaired individuals in the three cohorts, we favoured the use of previously published selection criteria for GENIC, NACC, and ADNI instead of conducting a hard post-hoc harmonization across cohorts for our current study. With this choice, we facilitate the comparison of our current findings with the extensive literature available from these three cohorts and increase generalizability of results.

Participants from all three cohorts underwent an extensive neuropsychological protocol. Only participants with complete data on the cognitive variables selected for connectome construction (see below) were included.

According to the Declaration of Helsinki, all individuals participated voluntarily and gave informed consent, after approval of local ethics committees.

### Cognitive variables and cognitive domains

The global cognitive assessments employed were the MMSE for GENIC and ADNI cohorts and the Montreal Cognitive Assessment (MoCA) for the NACC cohort. All other cognitive variables were classified into domains using the Diagnostic Statistical Manual (DSM)-5 for mental disorders (APA, [32]), as follows: complex attention, memory and learning, executive functions, perceptual-motor functions, and language.

Table 1 shows the final list of 47 cognitive variables for the GENIC cohort, 23 variables for the NACC cohort, and 24 variables for the ADNI cohort.

**Table 1:** Cognitive domains based on the DSM-5 and cognitive variables across cohorts

Cognitive Domains (DSM-5)	GENIC	NACC	ADNI
**Complex attention** (sustained attention, divided attention, selective attention, processing speed)	Digit Span Forward Spatial Span Forward Colors Trails Test PCV Reaction Time PCV Motor Time Stroop Words Stroop Colors	Digit Span Forward Trail Making Test Errors MoCA Trail Making Test	Trail Making Test A Time Trail Making Test A Errors Trail Making Test B Time Trail Making Test B Errors Digit Symbol Digit Span Forward
**Memory and learning** (Free and cued recall, implicit learning, semantic)	Logical Memory A Immediate Logical Memory B1 Immediate Logical Memory B2 Immediate Logical Memory A Delay Logical Memory B Delay Logical Memory A Recognition Logical Memory B Recognition TAVEC Trial 1 TAVEC Learning TAVEC Interference TAVEC Short Delay TAVEC Short Delay Clues TAVEC Long Delay TAVEC Long Delay Clues SRT Trial 1 SRT Learning SRT Interference SRT Short Delay SRT Long Delay	Benson Complex Figure Delay Benson Complex Figure Recognition Craft Story 21 Immediate Craft Story 21 Delay MoCA Registration MoCA Delayed Recall No Cue MoCA Delayed Recall Cue MoCA Recognition	AVLT Learning 1 AVLT Learning Tot AVLT Learning 1 Errors AVLT Learning Tot Errors AVLT B Interference AVLT B Errors AVLT Immediate AVLT Immediate Errors AVLT Delay AVLT Delay Errors AVLT Recognition AVLT Recognition Errors
**Executive functions** (Planning, decision making, working memory, responding to feedback, inhibition, flexibility)	Phonemic Verbal Fluency Stroop Inhibition Digit Span Backward Spatial Span Backward Hanoi Tower Trial 1 Hanoi Tower Learning Hanoi Tower Delay Action Verbal Fluency	Phonemic Verbal Fluency Phonemic Fluency Intrusions Digit Span Backward MoCA Abstraction MoCA Calculation Ser7 MoCA Letter A MoCA Orientation	Digit Span Backward Semantic Verbal Fluency Errors
**Perceptual- motor functions** (Visual perception, visuo-constructional reasoning, perceptual-motor coordination )	WAIS Block Design Facial Recognition Test Visual Reproduction I Tot Visual Reproduction II Tot Visual Reproduction Copy Visual Reproduction Recognition Judgement of Line Orientation I Judgement of Line Orientation II Luria’s HAM Right Luria’s HAM Left Luria’s Coordination	Benson Complex Figure Copy MoCA Visuoconstructive	Clock Test Copy Clock Test Tot
**Language** (Object naming, word finding, fluency, grammar and syntax, receptive language)	Boston Naming Test Semantic Verbal Fluency	Multilingual Naming MoCA Repetition Semantic Verbal Fluency	Boston Naming Test Semantic Verbal Fluency

AVLT, Auditory Verbal Learning Test; HAM, Hand Motor Alternations; MoCA, Montreal Cognitive Assessment; PCV, Personal Computer-Vienna System; SRT, Selective Reminding Test; TAVEC, Test de Aprendizaje Verbal España-Complutense

Before constructing the cognitive connectomes, we inspected the distribution and characteristics of all cognitive variables and performed the Kolmogorov-Smirnov test and QQ plots. We identified variables that did not follow a normal distribution (i.e., error variables) and transformed them into dummy variables as in previous publications [30]. Additionally, to facilitate results interpretation, cognitive variables were inverted when appropriate (i.e. time variables or error variables), so that higher values in all the included variables consistently reflected better performance. These steps ensured a uniform scoring direction and prevented spurious negative correlations that could otherwise alter network topology during binarization.

Furthermore, we accounted for the potential confounding effect of age and education by using multiple linear regression as described in Amato et al., [33]. Education was operationalized as years of schooling for NACC and ADNI cohorts. For GENIC, education was approximated using the total score of the Wechsler Adult Intelligence Scale (WAIS-III) Information subtest, as in previous GENIC studies (Garcia-Cabello et al., [17]). In the GENIC cohort, the WAIS-III Information subtest is a better proxy of educational attainment and benefit from schooling than years of schooling, due to the heterogeneity in educational backgrounds across different age groups in this cohort (Garcia-Cabello et al., [25]).

### Connectome construction and graph measures

Each cognitive variable was considered as a node in the cognitive connectomes. Edges between nodes were calculated as pair-wise Spearman correlation coefficients between all cognitive variables. In the NACC cohort, one variable (Trail Making Test Time) exhibited minimal correlations with the other cognitive variables. As our objective was to investigate connected cognitive connectomes, we excluded the Trail Making Test Time variable from further analyses. We retained both positive and negative correlations but excluded self-connections from the cognitive connectomes, as in previous publications [30, 34].

We calculated several network measures. Table 2 includes common definitions of all investigated network measures, with examples to facilitate interpretation through the manuscript and make the findings accessible to the reader. Briefly, we applied the Newman algorithm to the weighted cognitive connectomes to identify modules and qualitatively describe communities of cognitive variables [35]. For quantitative analysis, we calculated the following global network measures: global efficiency (measure of network integration), local efficiency (measure of segregation), betweenness centrality (measure of centrality), and modularity (measure of segregation and integration) [24]. We also calculated the following nodal network measures: global efficiency, local efficiency, and betweenness centrality for each node. The selection of these network measures allows characterizing the balance between integration and segregation features of the cognitive network, as illustrated in Table 2. Integration refers to the capacity of a cognitive connectome or network to transfer information efficiently throughout the network (as opposed to a fragmented network where performance in certain cognitive variables does not correlate with that in other cognitive variables). Segregation refers to the capacity of a cognitive network to include specialized sub-networks, for example, smaller groups of cognitive variables that belong to the same cognitive domain (e.g. memory, attention, etc.). A normal cognitive connectome free of alterations would keep a proper balance between integration and segregation features.

**Table 2:** Common definitions of network measures and examples for their interpretation

Network Measures	Formal definition	Interpretation	Low values	High values
**Global efficiency (Integration)**	Average of the inverse shortest path lengths between all node pairs	Reflects ease of information transfer across the entire cognitive network	Fragmented network with reduced capacity for communication across cognitive domains	Highly integrated cognitive network with efficient communication across cognitive domains
**Local efficiency (Segregation)**	Average efficiency of information transfer within small neighbourhoods	Indicates resilience and efficiency of communication within a specific cognitive domain	Reduced resilience within neighbourhoods	Resilient network where neighbouring cognitive functions are strongly inter-connected
**Betweenness centrality** **(Integration)**	Fraction of all shortest paths in the network that pass through a node	Identifies “bottleneck” or “hub” nodes that act as bridges between other cognitive nodes	Peripheral cognitive node with minimal influence on information flow	Crucial cognitive node that guides information flow in the network
**Modularity (Integration & Segregation)**	Degree to which the network can be partitioned into sub-networks (clusters / modules / cognitive domains)	Quantifies the balance between specialized clusters (e.g. cognitive domains) and overall network cohesion (e.g. between cognitive domains)	Overly integrated network with low distinction between cognitive domains	Highly partitioned cognitive network with specialized cognitive domains

All global and nodal network measures were calculated on unweighted binarized cognitive connectomes across a range of network densities. The range of densities ensured that the connectomes were connected (each node being connected to at least one other node, lower range limit) and showed small-world characteristics on the higher range limit (small-world index ≥ 1.2), as in previous publications [27]. This procedure was performed separately in each cohort, providing the following cohort-specific range of densities: 25% to 62% in the GENIC cohort, 50% to 70% in the NACC cohort, and 21% to 63% in the ADNI cohort, in steps of 1% in all cohorts.

### Statistical analysis

We conducted comparisons between men and women across demographic and cognitive variables. We applied *t*-tests for age and education. ANCOVAS were used for univariate comparisons of men and women across cognitive domains while controlling for age, education, or MMSE/MoCA scores as detailed in the results section. Cohen’s *d* was used to assess effect sizes, which were directly obtained from *t*-tests or converted from partial eta square statistics from ANCOVA. Cohen’s *d* was interpreted as small (*d* = 0.2), medium (*d* = 0.5), and large (*d* = 0.8) effect sizes. For all these analyses, significance was defined as *p*-value < 0.05.

Between-group comparisons of global network measures were conducted by comparing the observed differences in cognitive connectomes between men and women to group differences produced by 10,000 non-parametric permutations of the cognitive connectomes in the previously described ranges of network densities. To ensure the reliability of our results, we adapted the approach used in previous studies [27, 30], to the multi-cohort design in the current study. Specifically, we considered a finding meaningful if significant differences were observed in at least 25% of the densities within each cohort.

For nodal measures, we applied the False Discovery Rate (FDR) adjustment for multiple testing with a significance level of *p* ≤ 0.05 [36]. Similarly to the approach for global results, nodal results were considered significant if differences in at least 25% of the densities survived the correction for multiple testing.

All graph and statistical analyses were performed using the Brain Connectivity toolbox in MATLAB versions R2019b and R2023b.

## Results

### Demographic characteristics

Table 3 shows demographic and cognitive characteristics of men and women in the GENIC, NACC and ADNI cohorts. We did not find any statistically significant difference in age between men and women. In contrast, women had significantly lower education in the three cohorts. Women showed significantly higher MMSE scores than men in GENIC but lower in ADNI, after accounting for age and education.

**Table 3:** Demographic data and cognitive domains of groups in GENIC, NACC, and ADNI cohorts (corrected for age and education)

	GENIC			NACC			ADNI		
	Women (*N*=188) (m ± SD)	Men (*N*=146) (m ± SD)	Effect size	Women (*N*=2127) (m ± SD)	Men (*N*=1576) (m ± SD)	Effect size	Women (*N*=108) (m ± SD)	Men (*N*=114) (m ± SD)	Effect size
Age	58.1 ± 11.1	57.5 ± 11.5	0.056	72.09 ± 9.26	72.36 ± 9.07	- 0.030	76 ± 4.78	75.76±5.36	0.047
Proxy of education									
WAIS-III Information	13.6 ± 6.0	17.6 ± 6.0	-0.665***						
Years of Schooling				15.54 ± 2.92	16.61 ± 2.82	-0.373***	15.17 ± 2.84	16.73±2.64	-0.570***
Global cognitive performance									
MMSE	28.7 ± 1.4	28.3 ± 1.6	0.605***				29.20 ± 0.95	28.99±1.05	0.420**
MoCA				24.60 ± 3.07	24.99 ± 2.54	0.001			
Cognitive domain									
Complex attention	-0.10 ± 0.70	0.13 ± 0.75	0.043	-0.04 ± 0.63	0.05 ± 0.62	0.066*	-0.00 ± 0.56	0.00± 0.58	0.160
Memory and learning	0.05 ± 0.64	-0.07 ± 0.70	0.784***	0.01 ± 0.56	-0.02 ± 0.54	0.134***	0.08 ± 0.50	-0.07± 0.52	0.413**
Executive functions	-0.11 ± 0.57	0.14 ± 0.61	0.051	-0.02 ± 0.49	0.03 ± 0.43	0.016	-0.00± 0.75	0.00± 0.65	0.005
Perceptual motor	-0.09 ± 0.65	0.12 ± 0.75	0.338	-0.02 ± 0.81	0.03 ± 0.68	0.003	-0.04± 0.94	0.04± 0.81	0.110
Language	-0.10 ± 0.80	0.13 ± 0.94	0.290*	-0.08 ± 0.73	0.10 ± 0.67	0.046***	-0.16±0.89	0.15 ±0.71	0.207

Different proxies of education were available for the three cohorts, as follows: WAIS-III Information subtest for the GENIC cohort and years of schooling for the NACC and ADNI cohorts. The available variables of global cognitive performance were the MMSE for both the GENIC and ADNI cohorts and MoCA for NACC. Cohen’s d was used to assess effect sizes, directly obtained from *t* -test for age and education. All other effect sizes in the table (and *p*-values) come from independent ANCOVAS. For global cognitive performance and all composites for cognitive domains, effect sizes are represented as Cohen’s *d* converted from partial eta squared (ηp^2^). *P*-values represented with asterisks as follows: *=*p* < 0.05;**=*p* < 0.01,***;*p* < 0.001. Abbreviations: ADNI, Alzheimer’s Disease Neuroimaging Initiative; GENIC, Group of Neuropsychological Studies of the Canary Islands; m, mean; MMSE, Mini-Mental State Examination; MoCA, Montreal Cognitive Assessment; NACC, National Alzheimer’s Coordinating Center; SD, standard deviations; WAIS-III, Wechsler Adult Intelligence Scale

### Univariate analysis of cognitive performance

In the GENIC cohort, women had a higher performance than men in the domain of memory and learning after accounting for age and education. This difference remained statistically significant after including MMSE as an additional covariate. Furthermore, women showed a lower performance than men in the language domain when controlling for age and education, but this difference was no longer statistically significant when including MMSE as an additional covariate (Table 3).

In the NACC cohort, women had a higher performance than men in the memory and learning domain after accounting for age and education. Additionally, women showed a lower performance in complex attention and language domains. All these differences remained statistically significant after including MoCA as an additional covariate (Table 3).

In the ADNI cohort, women showed a higher performance in the memory and learning domain when compared with men and accounting for age and education. This difference remained statistically significant after including MMSE as an additional covariate (Table 3).

Despite the reported sex differences, effects sizes were generally small, with some isolated differences reaching moderate effect sizes (Table 3).

### Graph analysis – correlation matrices

**Fig. 1:**
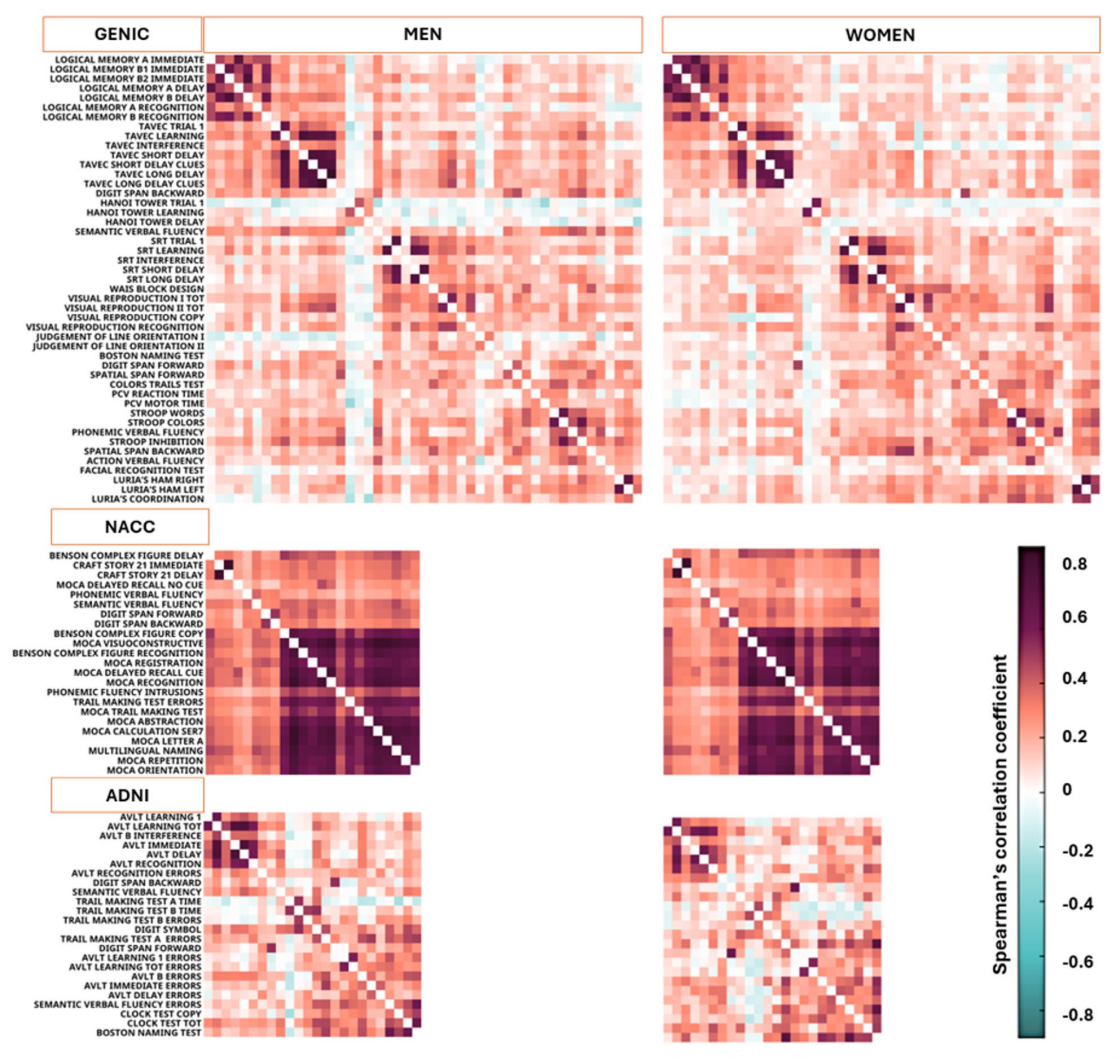
Cognitive connectomes for men and women across three cohorts based on Spearman’s correlations. The order of variables reflects the modular organization obtained for men in each cohort. The color bar reflects Spearman´s correlation coefficients, ranging from turquoise (negative correlation) to dark red (positive correlation). *AVLT*,* Auditory Verbal Learning Test; MoCA*,* Montreal Cognitive Assessment; MoCA SER7*,* Montreal Cognitive Assessment Serial 7; SRT*,* Spatial Recall Test; TAVEC*,* Test de Aprendizaje Verbal España-Complutense*

**Fig. 2:**
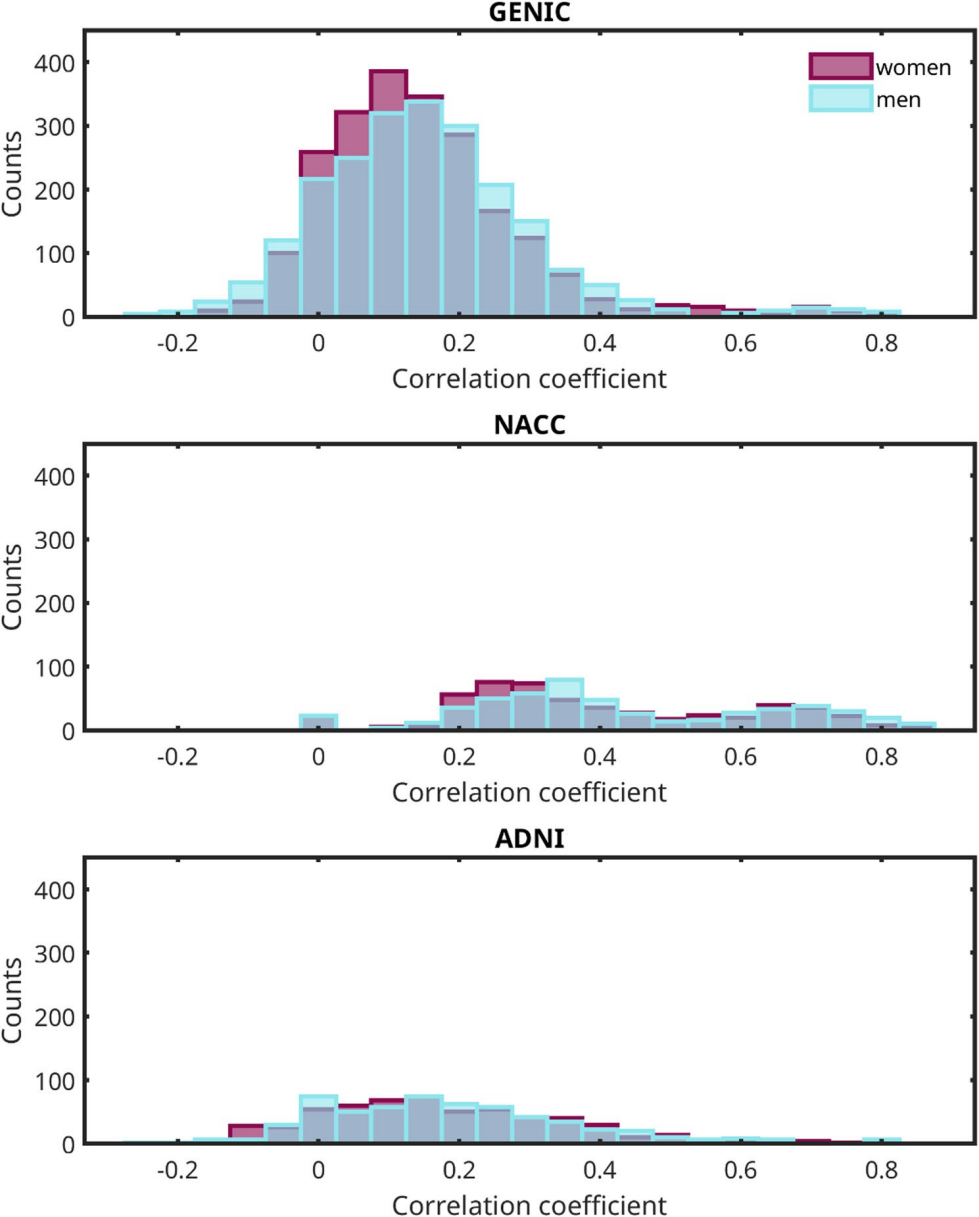
Distribution of correlation coefficients. Distribution (counts) of correlation coefficients across cognitive variables in women (purple columns) and men (blue columns) for the three cohorts

Figure 1 shows the cognitive connectomes of men and women in the three cohorts, where the visual inspection of matrices suggested a very similar network structure for both men and women (Fig. 1). While most correlations were positive, we observed 236 negative correlations for women (10.7%) and 290 for men (13.1%) in the GENIC cohort; 66 negative correlations in women (11.5%) and 60 in men (10.4%) in the ADNI cohort, and no negative correlations in the NACC cohort (Fig. 2). These negative correlations emerged despite uniform scoring direction where for all variables higher values consistently reflected better performance.

### Graph analysis – modular analysis

**Fig. 3:**
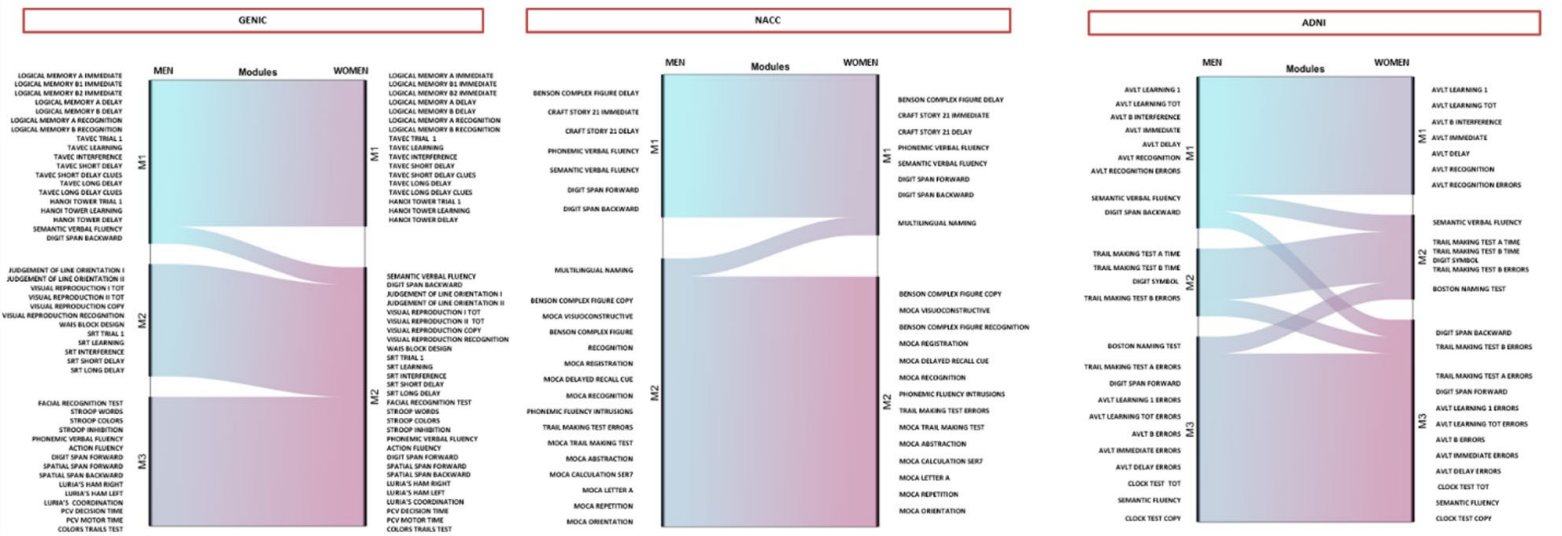
Alluvial plots. Alluvial plots illustrating cognitive variables included in each module for men (blue) and women (pink) across the three cohorts (modular allocation). *AVLT*,* Auditory Verbal Learning Test; MoCA*,* Montreal Cognitive Assessment; MoCA SER7*,* Montreal Cognitive Assessment Serial 7; SRT*,* Spatial Recall Test; TAVEC*,* Test de Aprendizaje Verbal España-Complutense*

Figure 3 shows the results from the modular analysis using the Newman algorithm (Fig. 3). Modular analysis allows examining how cognitive variables are related and cluster with each other, forming a cognitive connectome, and if the structure of the connectome differs between men and women. The modular structure or organization reflects the tendency of cognitive variables to cluster into partially separate (segregated) subsystems, each possibly supporting distinct cognitive functions.

Our results showed that, overall, the modular structure was very similar in men and women in GENIC and NACC, while it differed slightly in the ADNI cohort (Fig. 3).

In the GENIC cohort, women exhibited two modules and men showed three modules (Fig. 3). Module 1 included memory and executive functions and was largely the same in men and women, with the exception of semantic verbal fluency and digit backward span being assigned to module 1 in men and to module 2 in women. In women, module 2 encompassed several cognitive functions, including complex attention, executive functions, memory and learning, and perceptual-motor functions. In contrast, this module split into two modules in men: one module primarily included memory and learning variables along with perceptual-motor functions, while the third module grouped variables of complex attention, executive functions, and additional perceptual-motor functions.

In the NACC cohort, both sexes showed two modules with very similar test allocation (Fig. 3). Module 1 included supra-span memory and learning, executive control, and verbal fluency—reflecting higher-order cognitive functions. Module 2 included overall lower-order cognitive functions related to attentional control, visual and language abilities, and infra-span memory and learning variables. The only difference between sexes was the naming test being assigned to module 2 in men and to module 1 in women.

In the ADNI cohort, both sexes showed three modules but with slightly different modular organization. In women, module 1 encompassed learning and memory while in men, module 1 included learning and memory clustered with semantic fluency and executive control. Module 2 differed slightly across sexes, with variables of processing speed clustering together in men, while processing speed also clustered with semantic fluency and naming in women. In both sexes, most error variables clustered together in module 3.

### Graph analysis – quantitative analysis of network measures

**Fig. 4:**
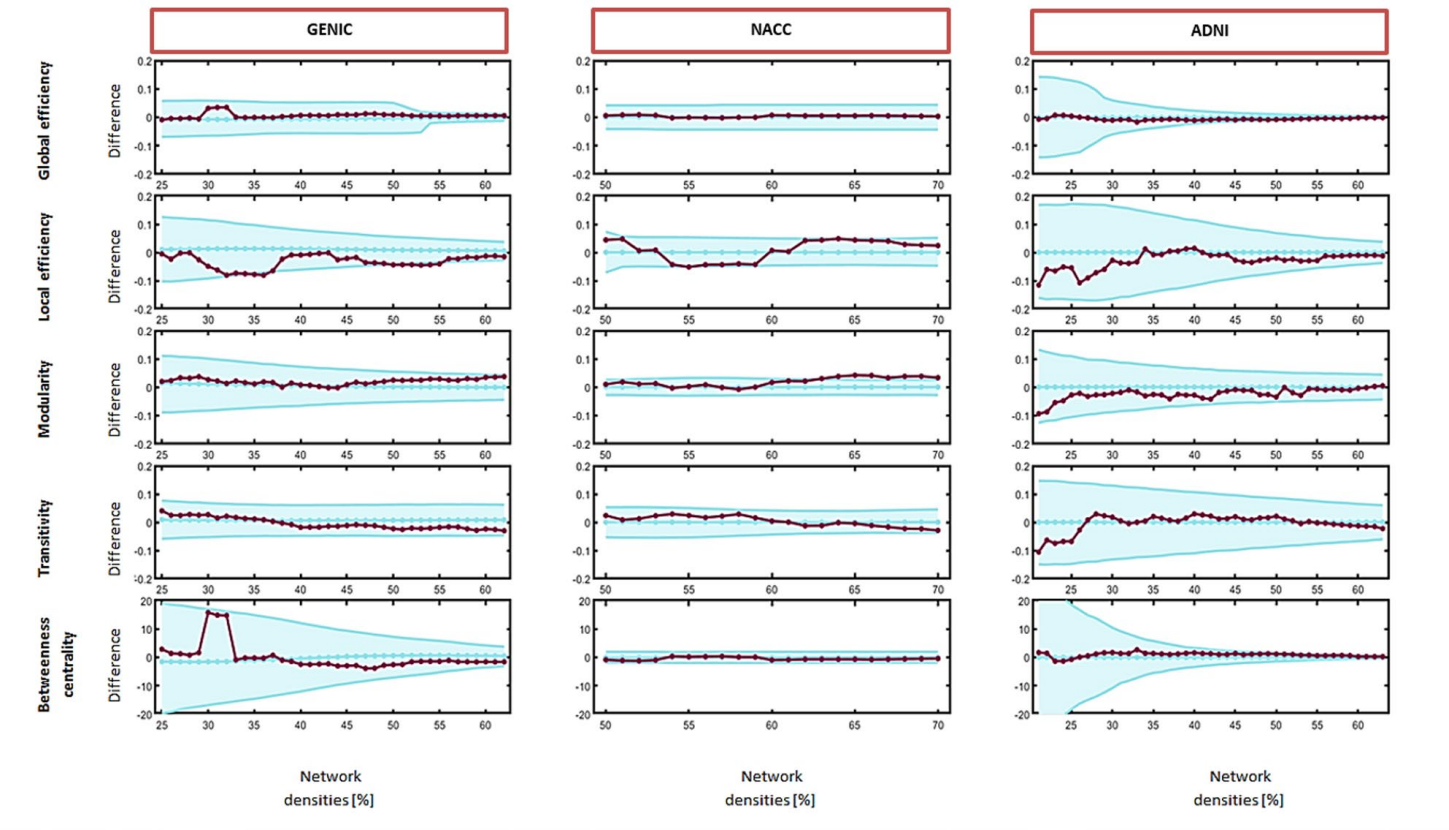
Sex differences in global network measures by cohort. Network densities are displayed on the x-axis, ranging from min = 20% to max = 60%, in 1% steps. Differences between men and women (purple points) are shown on the y-axis, with 95% confidence intervals of 10’000 permutations (blue area). Differences between groups are significant when the purple points are outside of the blue shaded area. Negative differences indicate lower value in men compared to women. Positive differences indicate higher values in women compared to men

Regarding global network measures, we found no statistically significant differences between men and women in any of the cohorts other than a higher modularity in women compared to men in the NACC cohort (Fig. 4). A higher modularity indicates a clearer segregation of the cognitive network in women, suggesting more specialized sub-networks (i.e. modules / cognitive domains).

In contrast, we observed several statistically significant differences in nodal graph measures (Table 4; Supplementary Table 1).

**Table 4:** Significant nodal network results by measure and cohort

Cognitive domains (DSM-5)	Cohort	Cognitive variables	Global efficiency	Local efficiency	Betweenness centrality
**Complex attention** (sustained, divided and selective attention, processing speed)	NACC	Trail Making Test Errors MoCA Trail Making Test			↓9
			↑6	↑17	↑17
	ADNI	Trail Making Test A Time Digit Symbol		↑33	↑33
			↓12		
**Memory and learning** (Free and cued recall, implicit learning, semantic)	NACC	Benson Complex Figure Delayed Benson Complex Figure Recognition Craft Story 21 Delayed MoCA Registration MoCA Delayed Recall No Cue MoCA Delayed Recall Cue MoCA Recognition		↓12	
			↑6		7↓
				6↓	
			↑13		
				↓10 / ↑9	↑21
					↓10
			↑5		↓8
**Executive functions** (Planning, decision making, working memory, responding to feedback, inhibition, flexibility)	GENIC	Hanoi Tower Trial 1 Hanoi Tower Learning		13↓	22↑
				13↓	
	NACC	Phonemic Verbal Fluency Phonemic Fluency Intrusions Digit Span Backward MoCA Abstraction		10↑ /11↓	18↑
			6↑	16↑	
				14↑	18↑
			6↑		
	ADNI	Digit Span Backward		↑13	↑12
**Perceptual Motor Functions** (Visual perception, visuo-constructional reasoning, perceptual-motor coordination )	GENIC	Facial Recognition Test			↑13
**Language** (Object naming, word finding, fluency, grammar and syntax, receptive language)	NACC	MoCA Repetition Semantic Verbal Fluency			20↓
				5↓	10↑ /11↓

↑ indicates higher values in women than in men; ↓ indicates lower values in women than in men. Numbers in the table indicate the number of consecutive density points within the analysed range of densities at which significant sex differences were observed. Only significant results are reported in the table, while blank cells reflect no statistically significant differences between men and women

In the GENIC cohort, women had a lower local efficiency than men in two executive variables and women had a higher betweenness centrality than men in one executive and one perceptual motor variable. Local efficiency reflects how information can be exchanged among closely related cognitive variables when a specific node (cognitive variable) is removed from the network. Therefore, a lower local efficiency indicates reduced resilience for those two executive variables in women. Betweenness centrality reflects the degree to which a cognitive variable is an important node in the network. Therefore, a higher betweenness centrality denotes a stronger role of that executive variable in women.

In the NACC cohort, we observed sex differences across all cognitive domains, except for perceptual-motor functions. Specifically, women showed higher global efficiency than men in variables related to memory and learning, executive functions, and complex attention. Global efficiency reflects the integration of the connectome – how easy information is transferred throughout the network. Therefore, those variables are better connected with the rest of the network in women. Women also had a higher local efficiency than men in executive functions and attention variables, and a higher betweenness centrality than men in four variables related to memory and learning, attention, and executive functions. However, these differences coexisted with a lower local efficiency in women than men in memory and language variables, and lower betweenness centrality in women than men in variables related to memory, attention, and language. Additionally, for one language-related variable, women displayed both higher and lower betweenness centrality than men depending on network density, higher at high densities and lower at low densities.

In the ADNI cohort, women had a higher local efficiency and betweenness centrality than men in complex attention and executive functions, concurrent with a lower global efficiency in women than men in complex attention.

## Discussion

We investigated sex differences in cognitive performance using both univariate and multivariate analyses, specifically, with the use of graph theoretical analyses on cognitive data. In univariate analyses, we found statistically significant sex differences of generally small effect sizes. Similarly, graph analyses revealed no major differences in cognitive correlation matrices, modular structure, and global network measures, although the nodal graph analyses demonstrated statistically significant differences in several cognitive variables.

The first aim of our study was to test for sex differences across five cognitive domains using a univariate approach. We observed statistically significant differences of small effect sizes in three cognitive domains, including memory and learning, language, and complex attention. Specifically, women showed a slightly higher performance than men in memory and learning in the three cohorts, and a slightly lower performance in the language domain in one cohort (ADNI) when accounting for age, education, and global cognitive performance. The higher performance of women in memory and learning may be attributed to their reported advantage in verbal episodic memory tasks, in line with previous univariate studies [7, 37]. The slightly lower performance observed in women in the language domain is consistent with prior univariate studies showing that men have a significantly higher performance than women on language tasks like the Boston Naming Test used in our study [38–40]. Additionally, only in the NACC cohort, women showed a lower performance in complex attention compared to men. This result aligns with recent studies suggesting that women tend to perform worse than men in sustained attention and visuospatial tasks [7, 41]. However, it is also worth noting that we could only include three variables in the complex attention domain for the NACC cohort, which may have contributed to the observed differences compared to the other two cohorts that included more variables of complex attention. All the reported sex differences in the univariate analyses showed small effect sizes, with some isolated differences showing moderate effect sizes.

Our second aim was to investigate sex differences using the multivariate approach of graph theory applied on cognitive data. The main results showed no major sex differences between men and women in cognitive correlation matrices, modular structure, and global network measures. When focusing on results at a nodal level, however, we observed several significant differences between men and women pinpointing some specific cognitive domains. We started with the visual inspection of correlation matrices and observed that they were largely comparable between men and women in the three cohorts. This suggests a similar cognitive organization in men and women, providing novel multivariate data to support the Gender Similarities Hypothesis by Hyde [21]. Furthermore, we observed a comparable number of positive and negative correlations in men and women across cohorts, again suggesting a similar cognitive organization in the two sexes. Next, we performed modular analyses and, again, we observed a similar modular structure across cohorts in men and women, except for the ADNI cohort. Specifically, both sexes exhibited three distinct modules in the ADNI cohort, but variable allocation to these modules was slightly different in men and women. The observed differential variable allocation suggests that men and women may use slightly different cognitive strategies to complete the same cognitive tasks, possibly underpinned by different structural connectivity as reported previously ([15]; Pletzer et al., [6]). It should be noted though that this finding was only observed in the ADNI cohort, but not in the GENIC or NACC cohorts. Therefore, we cannot exclude that this finding depends on the set of cognitive variables or population / cohort investigated.

In keeping with the overall lack of major sex differences in global features of the connectomes, the quantitative analyses of global network measures showed no statistically significant sex differences across cohorts, except for the higher modularity in women in the NACC cohort. Although this finding could suggest a more densely interconnected structure within modules in women, the higher modularity was observed only in the NACC cohort. Therefore, this finding could also be reflecting a more consistent performance of women across items of the MoCA test, since the connectome of NACC was heavily dependent on the MoCA test. While this quantitative measure of modularity extends the modular analysis reported above, the finding discussed in the previous paragraph for the NACC cohort was not replicated in this quantitative analysis of modularity. Therefore, we did not find any strong evidence indicating that neither modular allocation nor modularity are different between men and women and across the three cohorts. We would thus conclude for this part that the global features of the cognitive connectome of men and women seem to be very similar. As discussed below in the next paragraphs, this conclusion on global features contrasts with several differences captured on nodal features of the network.

The quantitative analyses of nodal network measures showed several statistically significant differences although with some variation across cohorts. Specifically, for the GENIC cohort, women had a lower local efficiency in two nodes of the executive domain along with a higher betweenness centrality in the executive domain and in visual perception (perceptual motor domain). These findings suggest that women present with a higher flow of information across domains, likely with the executive domain keeping a central role in distributing that information. Additionally, women may tend to recruit broader and more distributed networks when performing cognitive tasks. Of note, these processes would mostly operate at the local level or at the level of small subnetworks, without posing overt differences at the global network level as discussed above. These interpretations are consistent with previous evidence reflecting specific network strategies to offset reduced local efficiency by enhancing intermodular communication, thus maintaining the overall performance [42–44].

For nodal results in the ADNI cohort, women showed a lower global efficiency in sustained attention and a higher local efficiency and betweenness centrality in processing speed and executive functions. The lower global efficiency in sustained attention may reflect a lower integration of attention in the connectome, perhaps underlying the previously reported lower performance of women in this task ([5]; Pletzer et al., [6]). The higher betweenness centrality in processing speed and executive domains in women is consistent with the finding in the GENIC cohort.

For nodal results in the NACC cohort, we observed sex differences across all cognitive domains, except for perceptual-motor functions. While several of these results replicate those discussed for the GENIC and ADNI cohorts, the overall pattern in the NACC could also be related to methodological aspects, as a substantial number of cognitive variables in the NACC were items from the MoCA, which can increase the covariance among variables in the network [45]. Additionally, the large sample size of the NACC cohort likely contributed to the detection of subtle differences that may not emerge in smaller samples like GENIC and ADNI. With this in mind, the most consistent nodal results from the NACC cohort reinforce the interpretation of a central role of processing speed and executive functions for the distribution of information in women, and expose an additional finding suggesting that memory and learning and language domains may have a less important role in this context in women.

Integrating all the results from this study, the nodal network results showed an overall convergence with the sex differences captured by the univariate analyses. This observation does not suggest a one-to-one correspondence between univariate sex differences in task performance and nodal network differences in the cognitive connectome. Rather, both methods provide complementary information, with nodal network analysis extending beyond univariate performance effects. The nodal analyses helped characterize the complex association among multiple cognitive variables and the specific role of each cognitive node in the larger network or connectome. Furthermore, some nodal network differences emerged in domains where univariate analyses did not detect any significant sex differences, such as in executive and perceptual motor functions. These two cognitive domains rely on distributed neural systems involving multiple subcomponents, which may be better captured by network analysis than univariate analyses, the latter highlighting performance outcomes but not the network dynamics [46]. As a result, graph theory analyses detect nuanced differences in the structure and connectivity of networks beyond what is observable in univariate analyses [26, 44, 47].

Synthesizing all the results, while univariate analyses showed differences with small effect sizes, and global features of the connectomes were largely similar in men and women, nodal measures may reflect slightly different cognitive strategies operating in men and women when approaching specific cognitive tasks. In other words, our findings suggest that men and women may reach a similar global cognitive performance by approaching specific cognitive tasks slightly differently.

Several limitations should be acknowledged in this study. Firstly, the three cohorts differed in sample size, the number of cognitive tests included, and the criteria for defining and selecting cognitively unimpaired individuals. The three cohorts are also naturally different in cultural factors inherent in each cohort population. All these differences may have potentially introduced bias or inconsistencies when comparing results across cohorts. This heterogeneity may be particularly relevant for the interpretation of nodal-level results, since those depend more closely on the cognitive variables included. However, a closer inspection showed quite comparable procedures across cohorts and any residual differences and heterogeneity may also be considered a strength of the current study as we show largely consistent results across the three cohorts. Secondly, the lack of domain-specific variables for visuospatial abilities in the NACC and ADNI cohorts is another limitation, as visuospatial abilities are often reported to differ between men and women [7]. The GENIC cohort, which included enough domain-specific variables, may offer preliminary insights about sex differences in visuospatial components of the cognitive connectome that should be investigated further in future studies. Thirdly, the NACC dataset included a substantial number of variables from the MoCA, which may increase the covariance in the correlation matrices and under-represent some cognitive domains. The implications of this limitation can be partly observed by comparing the nodal results from NACC with those from GENIC and ADNI. Fourthly, while our study attempted to investigate sex differences, we did not explicitly account for gender identity, which could independently influence cognitive performance [48]. We acknowledge that some sex differences in cognition might reflect differences in lived experiences related to gender roles and expectations, rather than purely biological factors [49–51]. Finaly, the three investigated cohorts are primarily composed of White participants, which may limit the generalizability of findings. Although we corrected our cognitive variables for education, there may be residual effects on our data related to education, sociocultural, and health-related factors that vary across racial and ethnic groups and may interact with sex.

## Conclusions

In conclusion, using univariate analyses we demonstrated differences between men and women in cognitive performance with small effect sizes. While these differences were replicated and further characterized in nodal analyses using graph theory, these did not translate into differences in global features and modular organization of the cognitive connectomes of men and women. Based both on the previous research and the findings of our current study, we suggest that men and women may rely on slightly different cognitive strategies to approach specific cognitive tasks, while they achieve a largely comparable cognitive performance. This study contributes to the ongoing debate on cognitive sex differences by providing comprehensive data on connectome features supporting the idea of distinct strategies rather than inherent differences in cognitive ability.

## Supplementary Information

Supplementary Material 1.

## Data Availability

The authors of this study shall provide the generated dataset to encourage transparency and the ability to replicate research, upon reasonable request from qualified researchers. Requests for access to datasets should be directed to Daniel Ferreira (mail to: daniel.ferreira.padilla@ki.se).
