# Supplementary material for: The cognitive connectome of men and women: a study on sex differences across three cohorts
Source: Biol Sex Differ. 2026 Mar 14;17:69. doi: 10.1186/s13293-026-00866-0 (PMC13063598; doi:10.1186/s13293-026-00866-0)
Supplement: Supplementary file 1 — Supplementary Material 1. [file 13293_2026_866_MOESM1_ESM.docx]

**Supplemental Materials**

**Supplementary Table 1.** **Nodal network results by measure and cohort (full version)**

| **Cognitive domains**  **(DSM-5)** | **Cohort** | **Cognitive variables** | **Global efficiency** | **Local efficiency** | **Betweenness centrality** |
| --- | --- | --- | --- | --- | --- |
| **Complex attention**  (sustained attention, divided attention, selective attention, processing speed) | GENIC | Digit Span Forward  Spatial Span Forward  Colors Trails Test  PCV Reaction Time  PCV Motor Time  Stroop Words Stroop Colors |  |  |  |
|  | NACC | Digit Span Forward |  |  |  |
|  |  | Trail Making Test Errors |  |  | ↓9 |
|  |  | MoCA Trail Making Test | ↑6 | ↑17 | ↑17 |
|  | ADNI | Trail Making Test A Time |  | ↑33 | ↑33 |
|  |  | Trail Making Test A Errors |  |  |  |
|  |  | Trail Making Test B Time |  |  |  |
|  |  | Trail Making Test B Errors |  |  |  |
|  |  | Digit Symbol | ↓12 |  |  |
|  |  | Digit Span Forward |  |  |  |
| **Memory and learning**  (Free and cued recall, implicit learning, semantic) | GENIC | Logical Memory A Immediate  Logical Memory B1 Immediate  Logical Memory B2 Immediate  Logical Memory A Delay  Logical Memory B Delay  Logical Memory A Recognition  Logical Memory B Recognition  TAVEC Trial 1  TAVEC Learning  TAVEC Interference  TAVEC Short Delay  TAVEC Short Delay Clues  TAVEC Long Delay  TAVEC Long Delay Clues  SRT Trial 1 SRT Learning  SRT Interference  SRT Short Delay  SRT Long Delay |  |  |  |
|  | NACC | Benson Complex Figure Delay |  | ↓12 |  |
|  |  | Benson Complex Figure Recognition | ↑6 |  | 7↓ |
|  |  | Craft Story 21 Immediate |  |  |  |
|  |  | Craft Story 21 Delay |  | 6↓ |  |
|  |  | MoCA Registration | ↑13 |  |  |
|  |  | MoCA Delayed Recall No Cue |  | ↓10 / ↑9 | ↑21 |
|  |  | MoCA Delayed Recall Cue |  |  | ↓10 |
|  |  | MoCA Recognition | ↑5 |  | ↓8 |
|  | ADNI | AVLT Learning 1  AVLT Learning Tot  AVLT Learning 1 Errors  AVLT Learning Tot Errors  AVLT B Interference  AVLT B Errors  AVLT Immediate  AVLT Immediate Errors  AVLT Delay  AVLT Delay Errors  AVLT Recognition  AVLT Recognition Errors |  |  |  |
| **Executive functions**  (Planning, decision making, working memory, responding to feedback, inhibition, flexibility) | GENIC | Phonemic Verbal Fluency |  |  |  |
|  |  | Stroop Inhibition |  |  |  |
|  |  | Digit Span Backward |  |  |  |
|  |  | Spatial Span Backward |  | 13↓ | 22↑ |
|  |  | Hanoi Tower Trial 1 |  | 13↓ |  |
|  |  | Hanoi Tower Learning |  |  |  |
|  |  | Hanoi Tower Delay |  |  |  |
|  |  | Action Verbal Fluency |  |  |  |
|  | NACC | Phonemic Verbal Fluency |  | 10↑11↓ | 18↑ |
|  |  | Phonemic Fluency Intrusions | 6↑ | 16↑ |  |
|  |  | Digit Span Backward |  | 14↑ | 18↑ |
|  |  | MoCA Abstraction | 6↑ |  |  |
|  |  | MoCA Calculation Ser7 |  |  |  |
|  |  | MoCA Letter A |  |  |  |
|  |  | MoCA Orientation |  |  |  |
|  | ADNI | Digit Span Backward |  | ↑13 | ↑12 |
|  |  | Semantic Verbal Fluency Errors |  |  |  |
| **Perceptual Motor Functions**  (Visual perception, visuo-constructional reasoning, perceptual-motor coordination ) | GENIC | WAIS Block Design  Facial Recognition Test  Visual Reproduction I Tot  Visual Reproduction II Tot  Visual Reproduction Copy  Visual Reproduction Recognition  Judgement of Line Orientation I  Judgement of Line Orientation II  Luria’s HAM Right  Luria’s HAM Left  Luria’s Coordination |  |  | ↑13 |
|  | NACC | Benson Complex Figure Copy  MoCA Visuoconstructive |  |  |  |
|  | ADNI | Clock Test Copy  Clock Test Tot |  |  |  |
| **Language**  (Object naming, word finding, fluency, grammar and syntax, receptive language) | GENIC | Boston Naming Test  Semantic Verbal Fluency |  |  |  |
|  | NACC | Multilingual Naming |  |  |  |
|  |  | MoCA Repetition |  |  | 20↓ |
|  |  | Semantic Verbal Fluency |  | 5↓ | 10↑ 11↓ |
|  | ADNI | Boston Naming Test  Semantic Verbal Fluency |  |  |  |

Note: ↑ indicates higher values in women than in men; ↓ indicates lower values in women than in men. *Numbers in the table indicate the number of consecutive density points within the analysed range of densities at which significant sex differences were observed. Only significant results are reported in the table, while blank cells reflect no statistically significant differences between men and women.* Only significant results are reported in the table, while cells in blank reflect no statistically significant differences between men and women.
